# Financial Incapacity of Patients with Mild Alzheimer’s Disease: What Neurologists Need to Know about Where the Impairment Lies

**DOI:** 10.3390/neurolint14010008

**Published:** 2022-01-11

**Authors:** Vaitsa Giannouli, Magda Tsolaki

**Affiliations:** School of Medicine, Aristotle University of Thessaloniki, 54124 Thessaloniki, Greece; tsolakim1@gmail.com

**Keywords:** mild Alzheimer’s disease, financial capacity, cognitive function

## Abstract

Research in the last decade has focused on assessing financial capacity and incapacity mainly in old age, but new research has turned to address the question of how financial incapacity can be predicted by cognitive factors. The aim of this study was to identify which cognitive domains predict financial capacity and the relevant cognitive skills of patients with mild Alzheimer’s disease (AD) in order to assist neurologists in functional assessment and further patient referral. In this study, 109 patients diagnosed with mild AD were examined with a number of neuropsychological tests: Mini-Mental State Examination (MMSE), Functional Rating Scale for Symptoms of Dementia (FRSSD), Functional Cognitive Assessment Scale (FUCAS), Trail Making Test (TMT)-Part B, Rey-Osterrieth Complex Figure Test (ROCFT)-copy condition and delayed recall condition, Rey Auditory Verbal Learning Test (RAVLT), Boston Naming Test, Rivermead Behavioural Memory Test (RBMT), digit span forward and backward, WAIS-R digit symbol substitution test, Neuropsychiatric Inventory (NPI), Geriatric Depression Scale (GDS-15), and the Legal Capacity for Property Law Transactions Assessment Scale (LCPLTAS). LCPLTAS total score and relevant subdomains were best predicted only by the score of one item coming from MMSE: subtraction of serial sevens. This is the only measure of arithmetic testing in use for the Greek geriatric population. Financial capacity is severely impaired in the group of mild AD patients. In order to prevent financial exploitation cases, neurologists, neuropsychologists, psychiatrists, and geriatrists should pay close attention to the information from the relevant arithmetic question of MMSE, as it is one of the most widely administered screening tests in clinical settings.

## 1. Introduction

Alzheimer’s disease (AD)is often diagnosed in the mild dementia stage, which is characterized by significant cognitive decline that interferes with the independence of the person. So far, there has been little research focusing on the effects of possible comorbid depression in mild AD [1]. Research has demonstrated that loss of financial capacity is a common consequence of AD [2], but also of Parkinson’s disease [3], vascular dementia [4], frontotemporal dementia [5], and even amnestic Mild Cognitive Impairment (aMCI) [6]. For AD patients, there are data supporting the importance of written arithmetic skills, simple visuomotor sequencing, and immediate story recall for predicting financial capacity [7] as measured by Financial Capacity Instrument (FCI), which is based on the conceptual model developed by Marson [8]. One problem is that written arithmetic skills are not currently examined with the use of a standardized test for the older Greek population (thus rendering impossible the use of relevant neuropsychological tests in clinical practice in Greece). The only arithmetic test that is in use in the Greek geriatric population is the Mini-Mental State Examination (MMSE) item measuring subtraction of serial sevens. The patient is asked to count backwards by 7 starting with 100. One point is given for each correct answer, and the examiner stops after five answers (that is, 93, 86, 79, 72, 65). Executive function and verbal memory, which have been proven as secondary predictors, are included in this study along with other tests [7].

A similar research attempt has shown that measures of the central executive component of working memory (Wechsler Adult Intelligence Scale-Third Edition (WAIS-III) Digit Span Backward Subtest, Arithmetic, and Letter-Number Sequencing tests) are strongly correlated with the FCI domains (basic monetary skills, checkbook management, bank statement management, and bill payment) as well as the FCI total score, while a measure of the phonological loop component of working memory (WAIS-III Digits Forward) is not significantly correlated with any FCI subdomains or the FCI total score [9].

In this study, financial (in)capacity was examined following the approach by Marson, measuring both performance and judgment related to financial issues with many relevant subdomains, such as basic monetary skills, financial concepts, simple cash transactions, checkbook management, bank statement management, and financial decision making (e.g., inheritance/investment decisions) [8]. Financial capacity is thus considered to have many aspects and to include the assessment of a variety of activities and specific skills (such as arithmetic, counting coins/currency, paying bills, etc.) and judgment/decision-making skills [2,8]. The aim of this study was to examine in patients with mild AD whether the previously found cognitive predictors are important for financial capacity performance given the limited number of classic standardized neuropsychological tests that are in use in Greek clinical settings for older populations. 

## 2. Method

A total of 109 Greek participants ≥65 years old (61 women and 48 men) with a diagnosis of mild AD were included in the study. Neuropsychological evaluation was conducted by a clinical neuropsychologist as part of a pre-arranged visit at the Memory Clinic of Papanikolaou General Hospital and elderly day care centers in Thessaloniki, Greece. An extensive battery of neuropsychological tests was administered according to the routine protocol of administration at each site, lasting approximately 1 h, assessing all major cognitive domains (memory, language, attention and information processing speed, visual–spatial ability, and executive functions). A number of tests examining different cognitive functions were administered (see Table 1).

The scores of these neuropsychological tests are presented in more detail in [2], where data from healthy participants are also shown to be more than 2.5 SDs above the scores of mild AD patients in this current sample (see Table 2). The novelty of this study is that we used summary scores as well as scores regarding single items from the neuropsychological tests as input in the analyses. In addition to abovementioned, the 15-item Geriatric Depression Scale was given for diagnosing depression and as a measure of mood (cut-off 6/7 points) [21] (M_GDS-15_ = 1.31, SD_GDS-15_ = 1.53, minimum GDS score = 0 and maximum GDS score = 6). 

These neuropsychological tests were selected because their reliability, validity, sensitivity, and appropriateness have been established for the Greek population and because all of these tests had already been validated in a Greek population.

Financial capacity was assessed with the Legal Capacity for Property Law Transactions Assessment Scale (LCPLTAS) [2]. The LCPLTAS consists of seven main domains: (1) basic monetary skills, (2) cash transactions, (3) bank statement management, (4) bill payment, (5) financial conceptual knowledge, (6) financial decision making, and (7) knowledge of personal assets [2]. 

The exclusion criteria were as follows (the same as similar studies [2,3,4]): (1)age below 65 years, (2) being a non-native Greek speaker, (3) a recorded history of stroke, (4) a history of substance abuse or alcoholism, (5) thyroid disorders and/or diabetes, (6) concomitant serious medical illness and sensory deficits (significant visual and/or auditory impairment not corrected sufficiently by visual/auditory aids), (7) a history of other neurologic (e.g., previous and/or current traumatic brain injury, brain tumor, hydrocephalus, encephalitis, epilepsy and related neurosurgical interventions) and/or psychiatric disorders (such as schizophrenia or other affective disorder that may interfere with the patient’s neuropsychological performance). In addition to the above, (8) participants with a score of GDS greater than 6/7 were excluded as this is considered as a cut-off value for depression in the Greek older population [21]. 

The diagnosis of AD was determined by a consensus from a team of expert neurocognitive health professionals. To assess the participants, they did a neuropsychological examination, a neurological examination, and a neuropsychiatric assessment, as well as neuroimaging, such as computed tomography (CT) or magnetic resonance imaging (MRI), and blood tests when possible. All study participants provided written informed consent prior to their admission to the study, and in cases where the older adults could not give consent themselves, their caregivers did after a detailed description of the procedure. Caregivers had to be 18 years or older (in all cases, their age ranged from 40 to 60 years), a family member or privately hired caregiver who provides care, and able to give informed consent. All procedures were approved by the Ethics Committee of the Aristotle University of Thessaloniki (decision 2/27.3.2013) as part of a larger study [9] following the declaration of Helsinki. he study took place from March 2013 to April 2015.

## 3. Statistical Analyses

Statistical analyses were performed using the Statistical Package for the Social Sciences (SPSS) 26 (SPSS, Chicago, IL, USA). Characteristics of people with dementia were analyzed with descriptive statistics (mean, standard deviation, and frequency).

Pearson correlations were performed with all of the administered neuropsychological tests, demographic characteristics of the participants (age, education years), and LCPTLAS, while gender was controlled for in t-tests regarding neuropsychological performance. Linear regression analyses were performed with LCPTLAS as the dependent variable and the classic neuropsychological tests (which were previously found to be significantly correlated with LCPTLAS along with the important score for the subtraction of serial sevens item from the MMSE) as the independent variables. Each of the seven subdomains of LCPTLAS were used as dependent variables with all of the other neuropsychological tests as independent variables. Only raw scores were used regarding LCPTLAS performance as they are easy to use and produce during the neuropsychological assessment. Probability values < 0.05 (two-tailed) were considered statistically significant.

## 4. Results

Men and women did not differ in their demographic characteristics, such as age (t(107) = 0.333, *p* = 0.740), education years (t(107) = 0.581, *p* = 0.563) as well as their depression levels as measured by GDS (t(99) = 0.593, *p* = 0.554), and their overall cognition as measured by MMSE total score (t(98) = 0.451, *p* = 0.653).

Pearson correlations revealed that the LCPTLAS full scale total score correlated significantly with the years of education (r = 0.421, *p* < 0.001) a finding that is supported by prior relevant research [22], as well as with MMSE (r = 0.487, *p* < 0.001), RBMT delayed recall (r = 0.213, *p* = 0.036), Trail Making Test Part B (r = −0.494, *p* < 0.001), ROCFT-copy condition (r = 0.313, *p* = 0.002), WAIS-R digit symbol substitution test (r = 0.292, *p* = 0.011), and the arithmetic question score from MMSE (r = 0.750, *p* < 0.001).

The neuropsychological measures entered in the regression were those that correlated significantly with the LCPTLAS full scale, and they were used because they cover broad areas of cognitive functioning. Linear Regression model “Enter” method indicated that only the arithmetic item score from MMSE predicted LCPTLAS (R = 0.797; R^2^ = 0.635; see Table 3).

Among all of the MMSE items that were scored separately, only the arithmetic item score from MMSE was found to predict the subdomains of LCPTLAS. More specifically, only the arithmetic item score from MMSE predicted basic monetary skills (R = 0.769; R^2^ = 0.591), cash transactions (R = 0.753; R^2^ = 0.567), bank statement management (R = 0.764; R^2^ = 0.584), bill payment capacity (R = 0.772; R^2^ = 0.596), financial conceptual knowledge (R = 0.786; R^2^ = 0.618), financial decision-making (R = 0.787; R^2^ = 0.619), and knowledge of personal assets (R = 0.726; R^2^ = 0.528) (see Table 4). In order to eliminate Type 1 error, which occurs when a researcher incorrectly rejects a true null hypothesis, Bonferroni corrections were used by dividing alpha by the number of simultaneous predictor variables.

## 5. Discussion

The above results provide support for a noticeable financial incapacity of patients with mild Alzheimer’s disease when compared to healthy controls ([2]). In addition, the total MMSE score has been found to be of utmost importance in predicting financial incapacity in previous studies of different types and subtypes of neurocognitive disorders [2,3,4,5,6]. This study shows the importance of arithmetic tasks in financial capacity assessment. When a detailed neuropsychological examination is provided and records are kept for the response to each test item, we can infer a person’s financial (in)capacity based only on one item derived from MMSE: the score for the subtraction of serial sevens, which was often disregarded. In contrast to previous studies investigating patients with MCI and AD [7], none of the classic neuropsychological test scores (except for the abovementioned MMSE item) predicted financial incapacity in this sample of patients with mild AD. Although education in years has been found to influence LCPTLAS performance in older adults with aMCI [22], this is not confirmed in this group of older adults with a diagnosis of mild AD. 

Personalized neuropsychological assessment and emphasis on cognitive factors has been addressed for organizing relevant interventions and initiatives against financial abuse of older adults [23]. Additional biological factors have also been examined, showing that genes (such as Apoe ε-4) do not play a crucial role in predicting financial capacity in mild AD [24], but the volume of the left angular gyrus and amygdala do correlate significantly with financial capacity in MCI [25] as well as with the volume of the medial frontal cortex [26]. This relationship may be due to the cognitive functions that these areas serve such as language processing, number processing, spatial cognition, memory retrieval, and attention [25,26].

Although the current study has several strengths, some limitations exist. The sample consisted of patients with a diagnosis of mild AD, so generalizations to other stages and types of neurocognitive disorders is impossible. In addition to that, although depression was included as a possible confounder (depressive symptoms were measured by the GDS-15), apathy (which has been found to be important in financial capacity in Parkinson’s Disease with dementia and frontotemporal dementia [27]) was not measured, as it was not included in the standard examination protocol of the hospital.

This is the first study of its kind for the Greek geriatric population to show the importance of a single item from MMSE in predicting financial capacity performance in the group of patients with mild Alzheimer’s disease. The current results confirm previous findings on the detrimental influence of neurocognitive disorders on financial capacity, also found as incapacity in different patient groups, such as individuals with Parkinson’s Disease, Vascular Dementia, and aMCI [2,3,4,5,6]. In this study, there is an emphasis on the importance of MMSE as a useful tool in the assessments from the perspective of examining in more detail the participants’ performance on individual items. Prior studies revealed many important cognitive functions in predicting the management of bills, such as slowness, verbal fluency, executive functioning, and language, followed by age, gender, and diagnosis [28], while other researchers have shown that written arithmetic skills, simple visuomotor sequencing, and immediate story recall are important [7]. Regression in a sample of Greek older adults with dementia has already shown that tests such as MMSE, GDS, and Trail Making Part B can predict competence on LCPLTAS [2]. In all these cases, we have to keep in mind that the lack of corroborating results in the abovementioned studies may be due to the different measurements of financial capacity that were implemented and due to the different variables (neuropsychological tests) that were entered in the regression models as independent cognitive predictors.

These findings need to be replicated with other neuropsychological tests measuring similar cognitive domains, but this is difficult in Greece as there are no relevant norms for using parallel forms of tests, and there is a paucity of neuropsychological instruments in clinical practice [29,30]. Nevertheless, health professionals, including neurologists, should routinely target and demand further examination of arithmetic skills for assessment and intervention in older adults regarding financial capacity, especially if cognitive impairment is evident.

## Figures and Tables

**Table 1:** Neuropsychological tests and cognitive domains.

Cognitive Domains	Neuropsychological Tests
General cognitive function	Mini-Mental State Examination (MMSE) [10]
Activities of daily living	Functional Rating Scale for Symptoms of Dementia (FRSSD) [11]
Executive function	Functional Cognitive Assessment Scale (FUCAS) [12] Digit Span (WAIS-III) backward span [13] Trail Making Test (TMT)-Part B (time to completion; without taking into consideration the time limit as reported in the test manual, but the total time in seconds until completion) [14]
Visuospatial functions and memory	Rey-Osterrieth Complex Figure Test (ROCFT)-copy condition and delayed recall condition of the complex design [15]
Verbal learning	Rey Auditory Verbal Learning Test (RAVLT) [16]
Language	Boston Naming Test [17]
Episodic and everyday memory	Rivermead Behavioural Memory Test (RBMT) [18]
Motor speed, attention and visuoperceptual skills	WAIS-R digit symbol substitution test [19]
Attention	Digit Span (WAIS-III) forward span [13]
Neuropsychiatric symptoms	Neuropsychiatric Inventory (NPI) [20]

**Table 2:** Minimum–maximum, Mean and SDs regarding LCPTLAS total score and subscale scores.

Neuropsychological Tests	Minimum	Maximum	Mean	SD
LCPTLAS total score	0.00	212.00	145.74	73.64
Basic monetary skills subdomain	0.00	14.00	9.65	4.78
Cash transactions subdomain	0.00	8.00	4.88	3.27
Statement management subdomain	0.00	8.00	4.60	3.41
Bill payment subdomain	0.00	8.00	5.37	3.09
Conceptual knowledge subdomain	0.00	32.00	22.83	11.09
Decision making subdomain	0.00	114.00	77.62	40.52
Asset knowledge subdomain	0.00	28.00	20.77	9.10
MMSE	15.00	28.00	22.18	3.68
FUCAS	44.00	91.00	53.83	7.78
FRSSD	3.00	18.00	8.50	3.10
NPI	0.00	47.00	7.22	8.51
RAVLT immediate	0.00	7.00	3.28	1.62
RAVLT delayed	1.00	8.00	2.76	1.87
RBMT immediate	0.00	17.00	7.29	3.86
RBMT delayed	0.00	16.00	4.81	4.31
Trail Making Part B (secs to completion regardless of time limit)	185.00	962.00	440.22	222.00
ROCFT copy condition	2.00	36.00	23.03	8.87
ROCFT delayed recall	0.00	25.50	5.36	4.62
Boston Naming Test	10.00	52.00	30.24	10.39
WAIS-R digit symbol substitution test	3.00	34.00	14.56	7.63
Forward digit span	0.00	7.00	3.84	1.54
Backward digit span	0.00	9.00	2.78	1.73

**Table 3:** Regression of demographic and neuropsychological variables on LCPTLAS total score.

Predictors	Unstandardized Coefficients		Standardized Coefficients	t	*p*
	B	Std. Error	Beta		
(Constant)	27.886	44.540		0.626	0.534
Education in years	2.201	1.743	0.123	1.263	0.213
MMSE	2.489	1.530	0.147	1.627	0.110
RBMT delayed	0.819	1.184	0.064	0.691	0.493
Trail Making Part B	−0.033	0.028	−0.116	−1.188	0.241
ROCFT copy condition	0.444	0.997	0.046	0.446	0.658
WAIS-R digit symbol substitution test	−0.079	0.841	−0.010	−0.094	0.926
Arithmetic item from MMSE	30.379	4.417	0.651	6.878	<0.001
Dependent Variable: LCPTLAS total score					

Note: *p* values (<0.001) significant after Bonferroni correction.

**Table 4:** Neuropsychological predictors for each subdomain of LCPTLAS.

Predictors	B	Std. Error	Beta	t	*p*
Dependent Variable: Basic Monetary Skills					
(Constant)	2.083	2.987		0.697	0.489
Education in years	0.212	0.117	0.187	1.816	0.075
MMSE	0.115	0.103	0.107	1.119	0.269
RBMT delayed	0.098	0.079	0.120	1.231	0.224
Trail Making Part B	−0.002	0.002	−0.117	−1.127	0.265
ROCFT copy condition	0.059	0.067	0.096	0.882	0.382
WAIS−R digit symbol substitution test	−0.019	0.056	−0.039	−0.338	0.737
Arithmetic item from MMSE	1.703	0.296	0.576	5.749	<0.001
Dependent Variable: Cash Transactions					
(Constant)	−2.175	2.516		−0.865	0.391
Education in years	0.184	0.098	0.197	1.866	0.068
MMSE	0.146	0.086	0.166	1.693	0.097
RBMT delayed	0.111	0.067	0.167	1.660	0.103
Trail Making Part B	−0.001	0.002	−.080	−0.750	0.457
ROCFT copy condition	0.001	0.056	0.003	0.027	0.979
WAIS−R digit symbol substitution test	−0.006	0.048	−0.015	−0.128	0.898
Arithmetic item from MMSE	1.338	0.249	0.552	5.363	<0.001
Dependent Variable: Bank Statement Management					
(Constant)	−1.531	2.618		−0.585	0.561
Education in years	0.194	0.102	0.196	1.892	0.064
MMSE	0.108	0.090	0.116	1.205	0.234
RBMT delayed	0.131	0.070	0.186	1.885	0.065
Trail Making Part B	−0.002	0.002	−0.111	−1.059	0.295
ROCFT copy condition	−0.016	0.059	−.0029	−0.270	0.789
WAIS−R digit symbol substitution test	0.005	0.049	0.012	0.107	0.915
Arithmetic item from MMSE	1.452	0.260	0.564	5.591	<0.001
Dependent Variable: Bill Payment					
(Constant)	0.708	2.118		0.334	0.739
Education in years	0.104	0.083	0.129	1.260	0.214
MMSE	0.108	0.073	0.140	1.483	0.144
RBMT delayed	0.015	0.056	0.026	0.263	0.793
Trail Making Part B	−0.001	0.001	−0.102	−0.989	0.328
ROCFT copy condition	−0.0015	0.047	−0.033	−0.306	0.761
WAIS−R digit symbol substitution test	0.014	0.040	0.039	0.343	0.733
Arithmetic item from MMSE	1.351	0.210	0.640	6.434	<0.001
Dependent Variable: Financial Conceptual Knowledge					
(Constant)	1.087	6.884		0.158	0.875
Education in years	0.373	0.269	0.137	1.384	0.173
MMSE	0.390	0.236	0.152	1.651	0.105
RBMT delayed	0.016	0.183	0.008	0.090	0.929
Trail Making Part B	−0.004	0.004	−0.099	−0.991	0.327
ROCFT copy condition	0.217	0.154	0.147	1.406	0.166
WAIS−R digit symbol substitution test	−0.016	0.130	−0.013	−0.121	0.904
Arithmetic item from MMSE	4.285	0.683	0.607	6.277	<0.001
Dependent Variable: Decision Making					
(Constant)	15.250	25.461		0.599	0.552
Education in years	0.947	0.996	0.094	0.950	0.347
MMSE	1.459	0.874	0.153	1.668	0.102
RBMT delayed	0.335	0.677	0.047	0.494	0.623
Trail Making Part B	−0.018	0.016	−0.111	−1.105	0.275
ROCFT copy condition	0.137	0.570	0.025	0.241	0.810
WAIS−R digit symbol substitution test	−0.042	0.481	−0.010	−0.087	0.931
Arithmetic item from MMSE	17.356	2.525	0.664	6.874	<0.001
Dependent Variable: Knowledge of Personal Assets					
(Constant)	12,.464	5.380		2.317	0.025
Education in years	0.187	0.211	0.098	0.888	0.379
MMSE	0.163	0.185	0.090	0.883	0.381
RBMT delayed	0.113	0.143	0.083	0.788	0.435
Trail Making Part B	−0.005	0.003	−0.162	−1.457	0.152
ROCFT copy condition	0.060	0.120	0.058	0.498	0.621
WAIS−R digit symbol substitution test	−0.015	0.102	−0.018	−0147	0.883
Arithmetic item from MMSE	2.895	0.534	0.584	5.426	<0.001

Note: *p* values (<0.001) significant after Bonferroni correction.

## Data Availability

The data presented in this study are available on request from the corresponding author. The data are not publicly available due to privacy issues.
